# Changes in tail posture detected by a 3D machine vision system are associated with injury from damaging behaviours and ill health on commercial pig farms

**DOI:** 10.1371/journal.pone.0258895

**Published:** 2021-10-28

**Authors:** Richard B. D’Eath, Simone Foister, Mhairi Jack, Nicola Bowers, Qiming Zhu, David Barclay, Emma M. Baxter

**Affiliations:** 1 Animal Behaviour & Welfare, SRUC, Edinburgh, United Kingdom; 2 Innovent Technology Ltd, Turriff, Aberdeenshire, United Kingdom; 3 Garth Pig Practice Ltd, Driffield, Yorkshire, United Kingdom; Tokat Gaziosmanpasa Universitesi, TURKEY

## Abstract

To establish whether pig tail posture is affected by injuries and ill health, a machine vision system using 3D cameras to measure tail angle was used. Camera data from 1692 pigs in 41 production batches of 42.4 (±16.6) days in length over 17 months at seven diverse grower/finisher commercial pig farms, was validated by visiting farms every 14(±10) days to score injury and ill health. Linear modelling of tail posture found considerable farm and batch effects. The percentage of tails held low (0°) or mid (1–45°) decreased over time from 54.9% and 23.8% respectively by -0.16 and -0.05%/day, while tails high (45–90°) increased from 21.5% by 0.20%/day. Although 22% of scored pigs had scratched tails, severe tail biting was rare; only 6% had tail wounds and 5% partial tail loss. Adding tail injury to models showed associations with tail posture: overall tail injury, worsening tail injury, and tail loss were associated with more pigs detected with low tail posture and fewer with high tails. Minor tail injuries and tail swelling were also associated with altered tail posture. Unexpectedly, other health and injury scores had a larger effect on tail posture- more low tails were observed when a greater proportion of pigs in a pen were scored with lameness or lesions caused by social aggression. Ear injuries were linked with reduced high tails. These findings are consistent with the idea that low tail posture could be a general indicator of poor welfare. However, effects of flank biting and ocular discharge on tail posture were not consistent with this. Our results show for the first time that perturbations in the normal time trends of tail posture are associated with tail biting and other signs of adverse health/welfare at diverse commercial farms, forming the basis for a decision support system.

## Introduction

Farmers, veterinarians and scientists use informal or formal observations of animal behaviour to detect ill health [1] and other behaviours of welfare concern [2]. Automation of the detection and recording of animal behaviour is possible using technology including camera data processed by ‘machine vision’ methods [3]. This technology implemented on farm can become the basis for breeding programmes [4] or for real-time early warning of anomalous behaviour [5]. The application of machine vision and other sensor technologies, collecting data from machines, buildings and animals to provide early warning of anomalous events in real-time for management decision support is known as precision livestock farming (PLF) [6, 7]. Potential applications of PLF have been researched in the major livestock species including fish [8] poultry [9], sheep [10], cattle [11] and pigs [12].

Tail biting is an important problem behaviour in pig farming. Growing pigs bite each other’s tails, resulting in pain, infections and mortality [13–15]. It can be costly to deal with on-farm and carcase condemnation leads to lost revenue [16, 17]. In the EU and UK, producers are under increasing pressure to phase out tail docking, which (despite regulations) is still commonly used to reduce tail biting risk [18, 19]. Multiple risk factors affect tail biting including the provision of enrichment and substrates, as well as health, diet, space and competition, so there is no simple solution. Tail biting often occurs in unpredictable outbreaks, and this uncertainty can make farmers risk averse to try solutions other than tail docking [20].

In previous work we focussed on the potential of low tail posture to be used as an early warning sign of tail biting, as found by a number of other studies [21–25]. We kept intact-tailed pigs under conditions where tail biting was common and developed and validated a 3D camera machine vision system to measure tail posture [5, 26]. We found that the proportion of pigs with low tails increased pre-outbreak, peaking when fresh tail damage was highest and declining after measures were taken to prevent further tail injury (removing biters and injured pigs, adding enrichment).

Low tail posture could be a specific indicator of tail biting, but other evidence suggests that tail posture and movements might reveal pigs’ emotional state and thus their welfare more generally [27, 28]. This implies that experiences other than tail biting, which impact negatively on welfare (such as ill health or injury), could result in a reduction in high (or curly) tails and an increase in low tails.

To investigate two possible explanations for tail posture (tail injury, or poor welfare more generally) in this study we scored tail injuries, but also injuries due to ear [29] or flank biting [30], which are thought to be problem behaviours with similar causes to tail biting. Injuries due to biting as part of social aggression [31] were also scored. Finally we scored signs of lameness [32], and nasal and ocular discharge [33].

Many PLF studies including our own earlier work [5] show ‘proof of concept’ results, with relatively small numbers of animals or groups, studied for a limited period of time, and often with deliberate scientific control of the variables of interest (see discussion in [3]). As a stepping stone to commercial use in the pig industry, these technologies must first be further developed and proven to be applicable in the field on diverse commercial farms, and with ‘natural’ variation in the variables of interest (e.g. [9]). In particular, our previous work [5] had used intact-tailed pigs, while many commercial farms continue to have tail-docked pigs.

To that end, in this study we installed a 3D camera system over the feeders on seven diverse commercial grow/finish pig farms in the UK. 3D camera data analysed by machine vision software enabled pig weight and tail postures to be measured. We visited farms for regular tail injury and health scoring visits over a 17-month period, but all management decisions (feeding, housing, bedding, health interventions, moving pigs in and out etc.) were made by farm staff. UK farms are more diverse than in many countries, and farms we chose varied in location (Scotland, Yorkshire, Oxfordshire), genetics, building type (flooring, ventilation), pen size, type and quantity of enrichment, pig start and end weights, tail biting history and tail length (intact (undocked), varying length of docked tails).

Our aims were to determine whether tail posture varied in a predictable way over time within and between batches of pigs, and at different farms. We also aimed to identify whether deviations from these farm/batch/time trends would occur due to relationships between: 1) Tail posture and tail injury scores; 2) Tail posture and other signs of ill health or injuries caused by other problem behaviours.

## Materials and methods

### Ethical considerations

This study was approved in writing by the Animal Experiments Committee of Scotland’s Rural College (SRUC, https://www.sruc.ac.uk/research/research-operations/animal-ethical-committee/), approval number AE-BL 01–2019. The study was observational; there were no changes to the normal working practices of each farm, which were regulated by Scots and English Law (still followed the relevant EU directives) and in accordance with the Defra 2020 Code of Practice for the Welfare of Pigs [34]. During scoring, if any pig appeared to need veterinary care, project staff brought this to the attention of farm staff. Routine tail docking is not permitted in the UK, so farms that tail docked did so with written permission from, and under the advice of their veterinary practice in accordance with the law as summarised in the Defra Code of Practice [34].

### Animals and farms

Seven farms were recruited for this study. Five in Yorkshire, one in Scotland and one in Oxfordshire. Farm characteristics are summarised in Table 1. In total 1692 grower / finisher pigs in 41 production batches were the subjects of this study.

**Table 1:** Farm characteristics.

Farm location	Farm code	Enrichment	Floor	Tail length	Cameras	Pens	Batches	Mean pigs per batch	Pen area (m^2^)	Space allowance (m^2^/ pig)
**Yorkshire**	6	Other	Slats	Short dock	1	3	9	29	22	0.76
**Oxfordshire**	7	Straw	Concrete	Mid dock	1	2	7	14	15	1.07
**Yorkshire**	8	Straw	Concrete	Undocked	2	1	2	186	353	1.90
**Yorkshire**	10	Straw	Concrete	Undocked	1	1	3	165	227	1.38
**Yorkshire**	11	Other	Concrete	Mid dock	1	1	3	90	85	0.94
**Yorkshire**	13	Other	Slats	Short dock	1	1	3	36	25	0.69
**Scotland**	14	Straw	Slats	Undocked	1	4	14	9	11	1.22

‘Other’ enrichment refers to farms that had plastic (artificial) enrichment items, or a mixture of artificial and natural items. Tail length of mid dock means 1/2 or more of the tail remains, short dock was where 1/3 of the tail or less remains. Cameras refers to the number of cameras per pen, Pens refers to number of pens with cameras, and batches refers to the different unique groups of pigs that provided project data over time.

### 3D camera data collection

Each pen had a 3D camera made to our specifications (320 x 240 pixel resolution; IP67 water and dustproof rating) by Peacock Technology Ltd, Stirling, UK (https://www.peacocktech.co.uk/). These cameras use ‘time-of-flight’ technology to detect the distance at each pixel by sending pulses of infrared light from an LED and using the time delay between the pulse and its return to measure depth at each pixel. The cameras were mounted at a height of 2.0m, located above feeders and pointing down to cover an area of the pen approximately 3.0m x 2.5m. On one of the Yorkshire farms, a second camera was placed over the drinkers (Table 1). Ethernet data cables (Cat 5e) connected each camera to an industrial fan-less PC (http://www.fit-pc.com/web/products/fit-pc4/) that processed the raw ‘point cloud’ camera images using machine vision software, and uploaded this processed data to servers at Innovent Technology Ltd via a 4G data connection.

Innovent Technology Ltd’s proprietary QScan technology was used to locate and orient pigs from the ‘point clouds’. For each pig that was present under the camera and standing up, a weight algorithm estimated the pig’s weight from its body 3D volume, and a tail posture algorithm (a refined version of our previously system validated at 74% accuracy [5]) located the tail and measured its angle relative to the body. This angle is measured at the base of the tail so measurement is effective with docked and undocked tails. If a tail hangs down or is tucked against the body, a 0° angle is detected. If the tail protrudes, the algorithm measures its angle from 0–90°. The system functioned at a group level so tail detections cannot be assigned to individual pigs.

### Behaviour, health and injury scoring

On-farm scoring was carried out by four experienced members of staff who trained each other, comparing results to ensure consistency. Two scorers carried out the bulk of scoring on their own, (31.3% each), and trained the other two (23.4%, 9.4%), with the remainder of scoring being done jointly by one of the two principal scorers in combination with one of the others during training (3.1%, 1.6%). For reasons of biosecurity (and later Covid-19), as we depended on permission and goodwill of commercial farmers, visits with more than one scorer were kept to a minimum necessary for training. Consequently side-by-side quantification of inter-observer reliability was not possible, but the 4 scorers and 2 combinations of scorers were treated as 6 scorer categories for analysis. On farm scorers were always blind to 3D camera data when scoring. Farm visits took place at mean (±s.d.) intervals of 14.0±10.1 days. The mean (±s.d.) batch length was 42.4±16.6 days, and pens were scored a mean of 4.5±1.3 times.

At each visit, pig injuries and health were scored. To keep track of which pigs had been scored, a marker pen, spray or crayon (following farm practice) was used. Every effort was made to score every pig in the pen, but if pigs were very flighty, and in larger groups in pens, this sometimes fell to around 90% of pigs. Individual pigs were not ear tagged, and could not be identified between repeat visits, so although scoring was at an individual pig level, proportions of scores at the pen level were used in analysis.

Tail injuries were scored for severity (0–4), freshness (0–5), length loss (0–3; scored as a change from the expected length, compared by eye with unaffected pen-mates; which allows for undocked tails and different docking lengths), swelling (0–1); Flank injuries were scored for severity (0–3), freshness (0–4) and size (0–2). Ear injuries were scored separately for left and right ears for severity (0–3), freshness (0–4), size (0–3) and swelling (0–2). Body lesions on the neck and shoulder due to fighting were scored (0–2). Scores were given for Lameness (0–2), Nasal discharge (0–1), Ocular discharge (0–1). A score of zero always meant ‘not present’, with higher scores for increasing magnitude. The scoring systems are given in detail in S1 File.

### Camera data processing

The median (IQR) daily image count was 1971 (3769.75; range 21 to 56,411). The number of images captured was dependent on several factors, including the number of pigs in the pen, the camera height, the number of cameras in the pen, and cleanliness of the camera. For the Yorkshire farm that had 2 cameras in one pen, data were combined for each day. Days with fewer than 30 pig detections were discarded from subsequent analysis, as we considered that data were too sparse to be reliable.

The raw pig detections in 3D camera data for each pen were processed to generate 24hr summaries for analysis: For tail posture, the frequency of tail detections that had 0° tail angle (Low tails), 0.01° – 45° tail angle (Mid tails) or 45.01° – 90° tail angle (High tails) were used to calculate a daily proportion of each. In previous work [5] we had analysed the proportion of 0° (low tails), but here we decided to split the remaining pigs into mid and high for analysis. Overall 47% of detections were low, 21% were mid and 32% were high.

Pig batches had an average weight (measured with 3D cameras) of 45.7kg (sd = 13.8) when scoring started and were an average weight of 75.0kg (sd = 20.13) by the last scoring event.

### Injury score data summaries and processing for analysis

**Fig 1:**
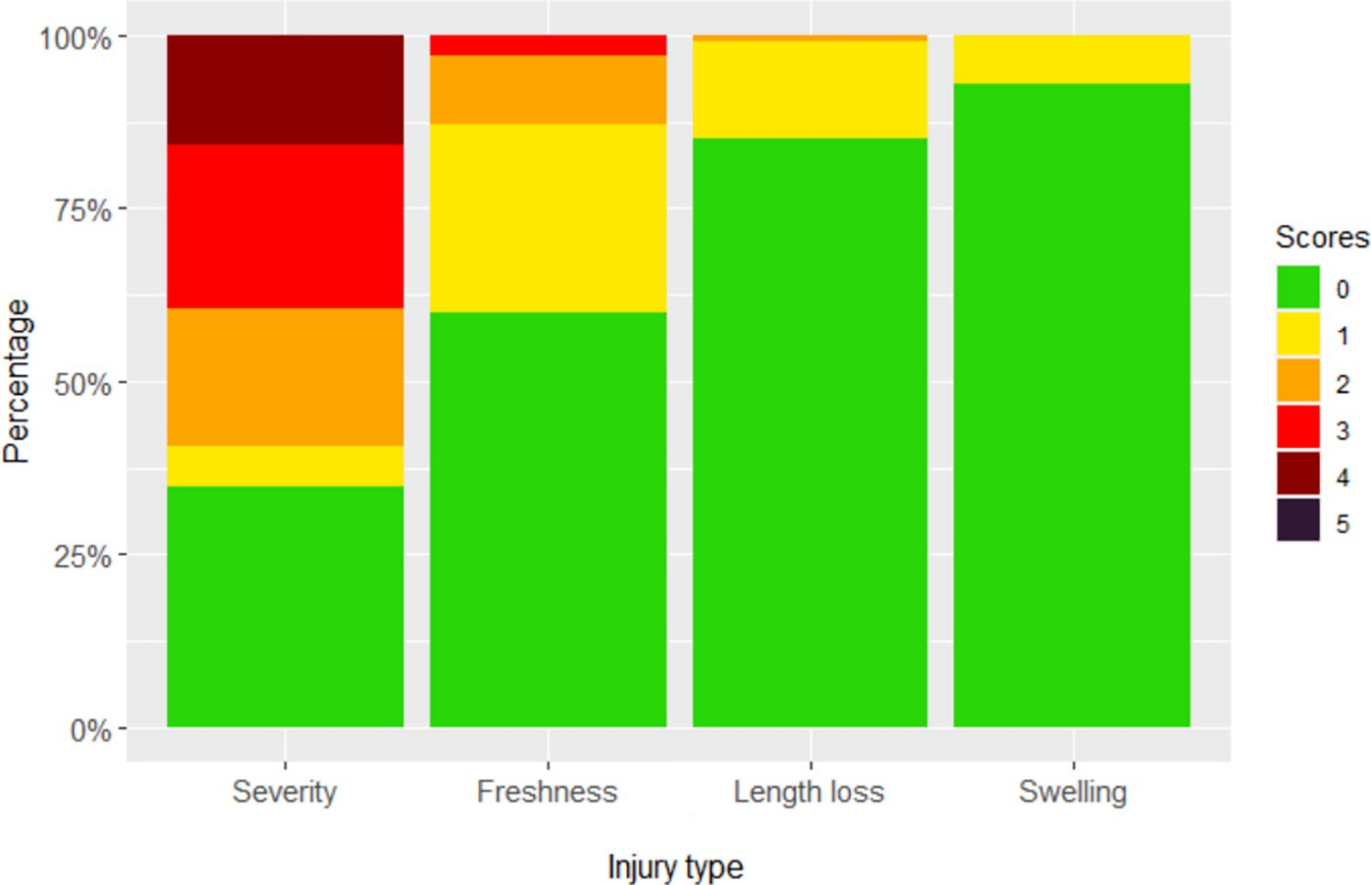
Stacked bar graphs of frequency of tail injury scores. Tails were scored on severity (0, 34.9%; 1, 5.6%, 2, 19.6%; 3, 23.6%; 4, 16.4%), freshness (0, 59.8%; 1, 27.1%; 2, 9.8%; 3, 3%; 4, 0.3%; 5, 0.1%), length loss (0, 85.4%; 1, 13.5%; 2, 0.8%; 3, 0.3%), and presence of swelling (0, 93.2%; 1, 6.8%) (See S1 File for score descriptions).

Across the different forms of injury scored, uninjured pigs were most common, and severe injuries were rare. Fig 1 shows the distribution of the tail injury scores resulting from tail biting. Severity scores of 2 (redness) and 3 (scratches or puncture marks) suggesting some minor tail biting were quite frequent, affecting over 40% of pigs (Fig 1). Wounds (score 4) were quite rare. For analysis, these data were combined into low tail injury severity (1 and 2 –flattened or red tails) and high tail injury severity (3 and 4 –indicting scratches, punctures or wounds). For scores of 3 or higher, non-zero values of tail injury freshness were scored. Fresh injuries were much rarer than scabbed injuries Combined categories were used for analysis—low freshness (1 and 2 indicating scabs) and high freshness (3, 4 and 5 indicating fresh, weeping or bleeding injuries). Fresh bleeding tails (5) were very rarely observed (0.1% of scored pigs). Non-zero values of tail length loss and tail swelling were both rare, so were converted to 1/0 scores for analysis (Table 2).

**Table 2:** Summary of how scores were combined to generate variables used in modelling.

Injury / health	Modelling variable name	Formatting method	Scores that were combined to form this new variable for modelling
*Tail*	Low tail injury severity	Grouped	Tail severity score 1 and 2
	High tail injury severity	Grouped	Tail severity score 3 and 4
	Low tail injury freshness	Grouped	Tail freshness score 1 and 2
	High tail injury freshness	Grouped	Tail freshness scores 3 and 4
	Tail length loss	Binary	Tail length loss score 1 to 3
	Tail swelling	Binary	Tail swelling score 1
*Flank*	Flank injury severity	Binary	Flank injury severity scores of 1 to 3
*Ears*	Low ear injury severity	Grouped	Ear injury severity score 1 and 2
	High ear injury severity	Grouped	Ear injury severity score 3 and 4
	Low ear injury freshness	Grouped	Ear injury freshness score 1 and 2
	High ear injury freshness	Grouped	Ear injury freshness score 3 and 4
*Lameness*	Lameness	Binary	Lameness 1 and 2
*Eyes*	Ocular discharge	Binary	Ocular discharge 1
*Body*	Body lesions	Grouped	Body lesion score 1 or 2 (discard 3 –used for dirty pigs)

**Fig 2:**
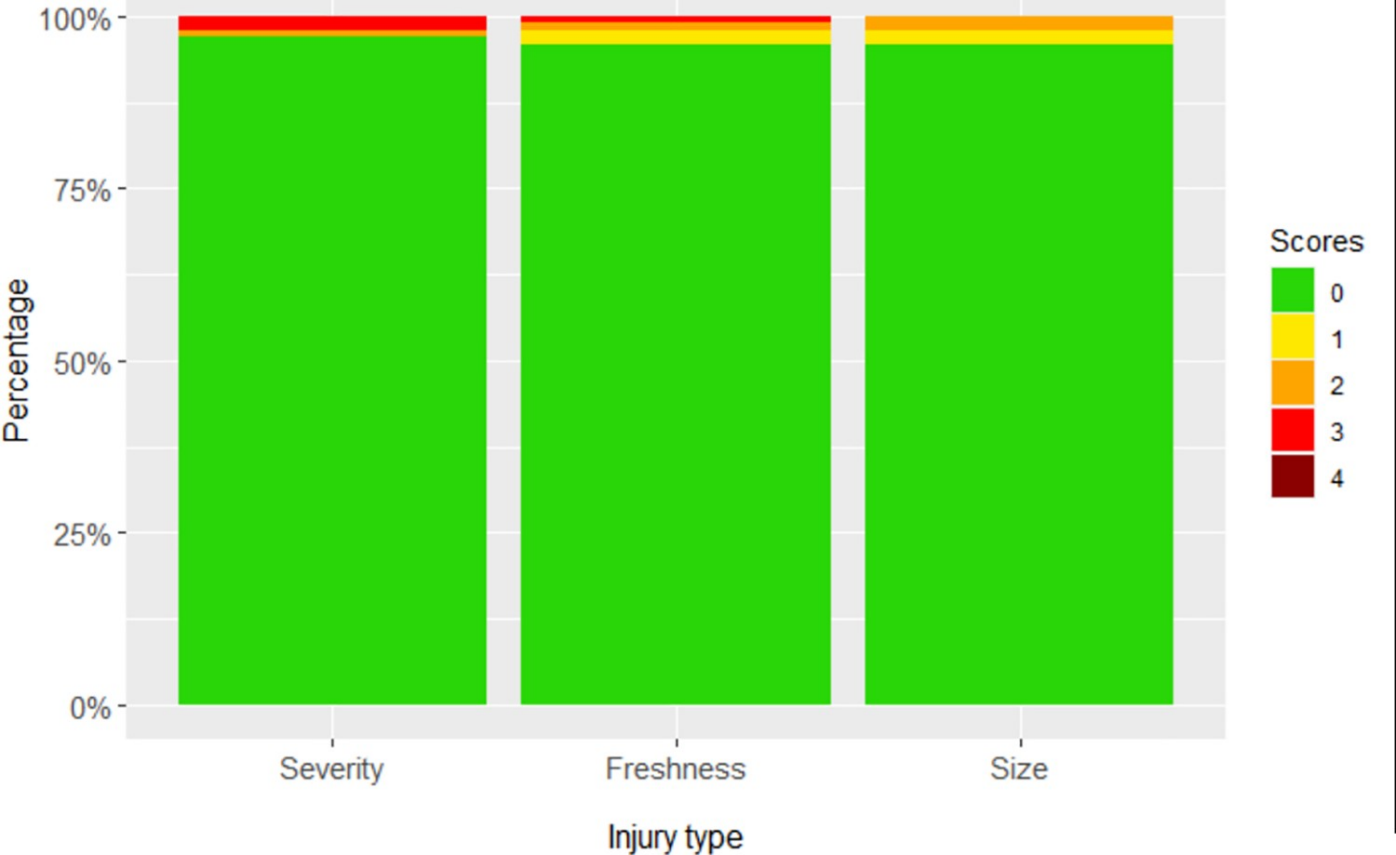
Stacked bar graphs of frequency of flank injury scores. Severity (0, 96.2%; 1, 0.3%; 2, 1.1%; 3, 2.4%), freshness (0, 96.2%; 1, 1.9%; 2, 1.2%; 3, 0.5%; 4, 0.1%) and size (0, 96.4%; 1, 1.7%; 2, 1.9%) of flank biting lesions are shown. (See S1 File for score descriptions).

Flank injury caused by flank biting was rare, with over 95% of pigs scoring zero meaning they had no sign of flank injury (Fig 2). Of those that did show flank injuries, scores were evenly distributed between 1–3. Flank injury scores of 1–3 were therefore combined, resulting in a 1/0 metric. Flank injury severity was highly correlated with flank injury freshness (ρ = 0.98, p<0.001) and size (ρ = 0.94, p<0.001), as such these were excluded from further analysis (Table 2).

**Fig 3:**
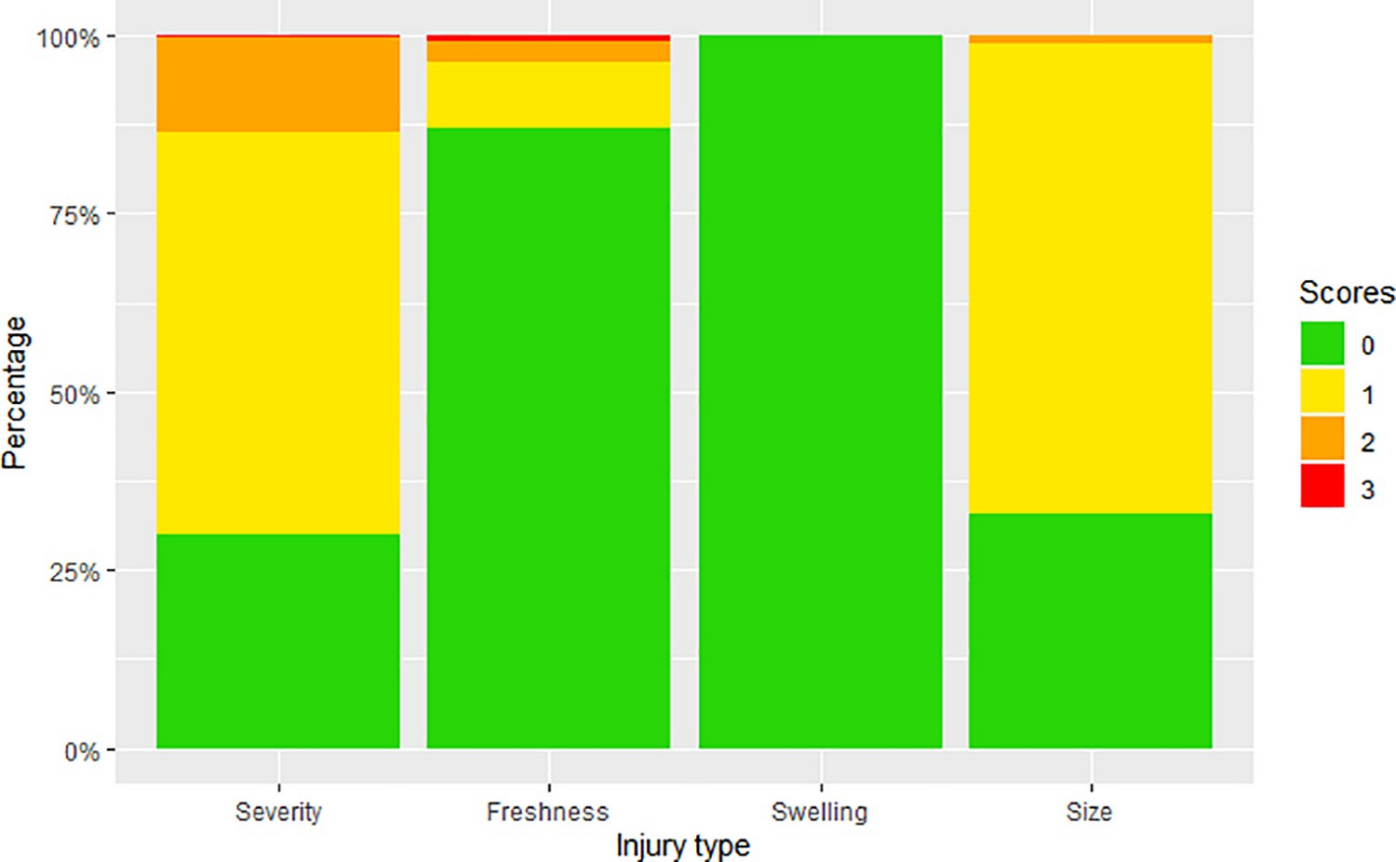
Stacked bar graph of frequency of ear injury scores. Severity (0, 30.0%; 1, 56.4%; 2, 13.4%; 3, 0.2%), freshness (0, 86.9%; 1, 9.2%; 2, 3.0%; 3, 0.9%; 4, 0.0%), swelling (0, 99.8%; 1, 0.1%; 2, 0.1%) and size (0, 32.8%; 1, 66.8%; 2, 1.2%; 3, 0.0%) of ear injury are shown. (See S1 File for score descriptions).

For ear injury caused by ear biting, a mean of the left and right ear scores was first taken and rounded up to the nearest integer value to result in a single value for each of severity, freshness swelling and size (Fig 3). Only 30% of ears had no signs of injury (severity 0), with minor injury (1, redness or scratches) in just over half of all pigs scored, and over 10% with damaged skin or wounds (severity 2 or 3). As for tail injury, ear injury severity and freshness were each categorised as either low (scores 1 and 2) and high (scores 3 and 4) for analysis. Ear injury size was found to be highly correlated with low ear injury (ρ = 0.96, p<0.001) and ear swelling was very rare so both were excluded from further analysis.

**Fig 4:**
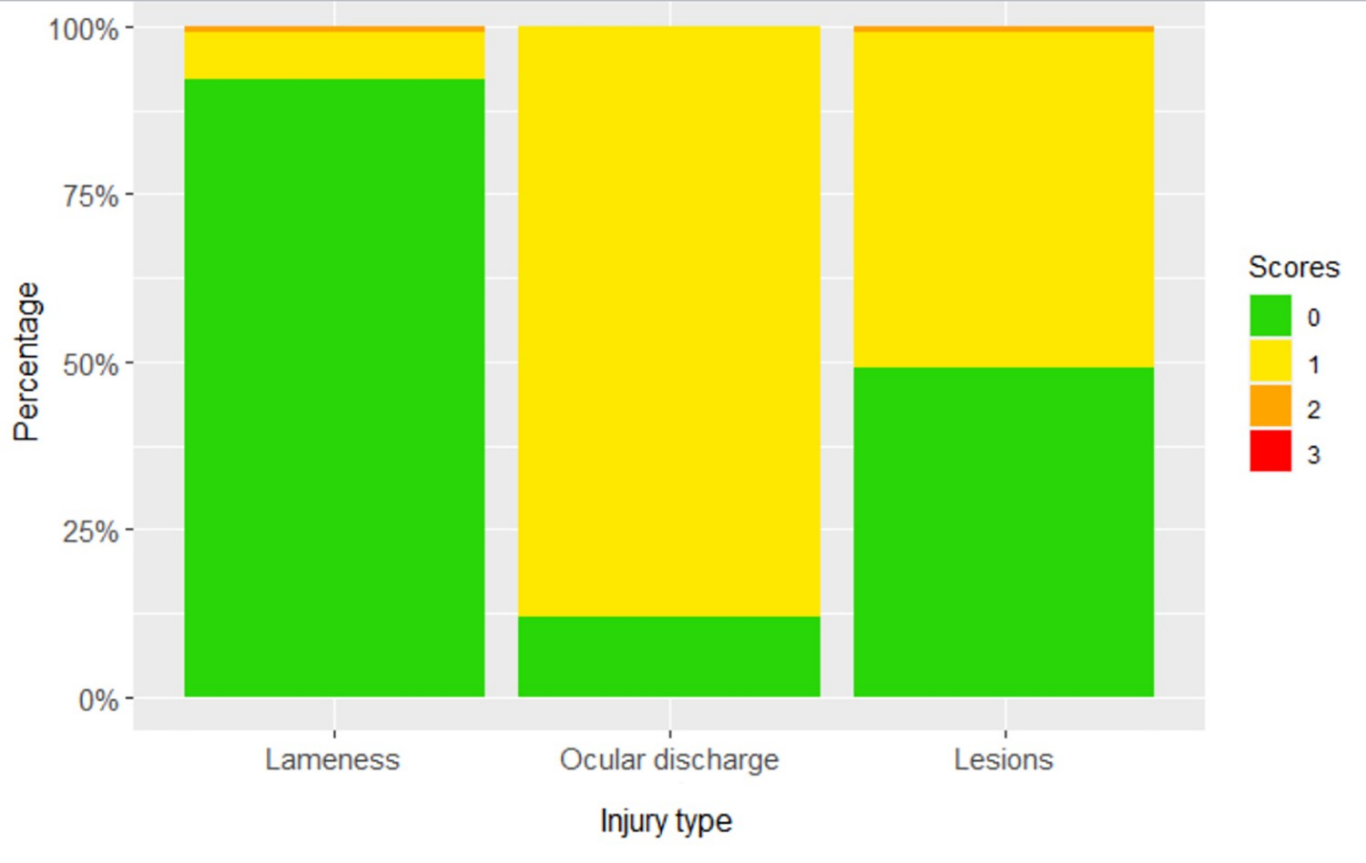
Stacked bar graph of frequency of other health scores. Lameness (0, 91.9%; 1, 7.0%; 2, 1.1%), Nasal discharge (0, 99.9%; 1, 0.1%), Ocular discharge (0, 12.3%; 1, 87.7%) and skin lesions resulting from social aggression (0, 49.1%; 1, 49.6%; 2, 0.9%; 3, 0.4%) (See S1 File for score descriptions).

Fig 4 shows the frequency of other health scores. Pigs showing any signs of lameness were rare (<10%), so lameness scores of 1 and 2 were combined for analysis (Table 2). Nasal discharge was observed only three times, so was excluded from further analysis. Ocular discharge was found in over four fifths of pigs scored. Skin lesions due to fighting ‘body marks’ were relatively common, with just over half of pigs having at least mild body marks (score 1) or more. Score 3 was used for pigs that were too dirty for body lesions to be scored. For analysis, scores of 1 and 2 were combined and 3 discarded, making this a 0/1 trait (Table 2).

### Variables used in modelling

**Fig 5:**
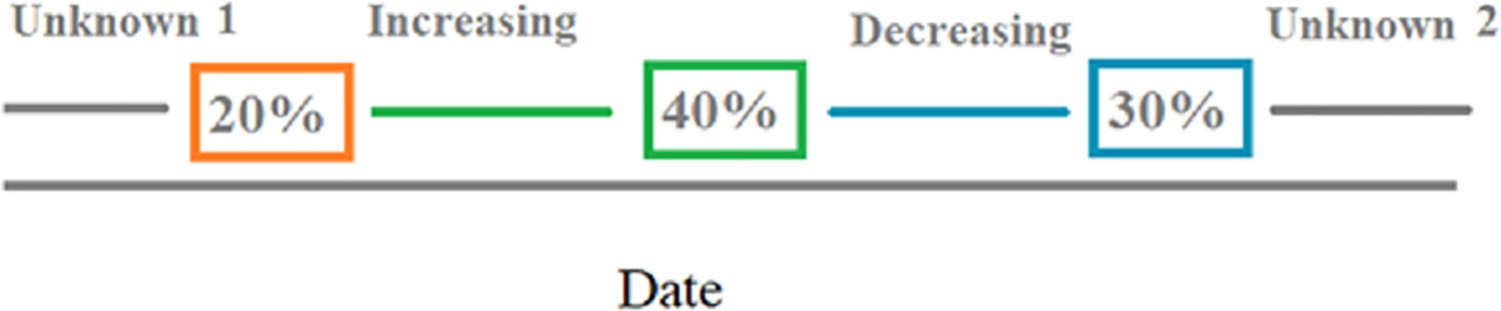
Example of categorisation of scoring periods as either increasing or decreasing tail injury. The percentages 20, 40 and 30 are included for illustration purposes to show the weighted total tail injury severity, and how changes in this metric would lead to a period being classified as increasing or decreasing. E.g. from 20% to 40% is an increase, while 40% to 30% is a decrease.

Based on the frequencies of scores shown in Figs 1–4, and correlations between scores, decisions on how to use the variables in modelling were made and summarised in Table 2. In addition, a weighted measure of overall tail injury scores was calculated. This was the sum of all tail injury severity scores as a percentage of the theoretical maximum (number of pigs in batch x maximum tail injury severity score 4). This weighted measure was used in analysis and was also used to generate a further variable used in analysis–a categorical variable to classify whether increasing or decreasing tail injury had occurred since the last scoring period (Fig 5).

**Fig 6:**
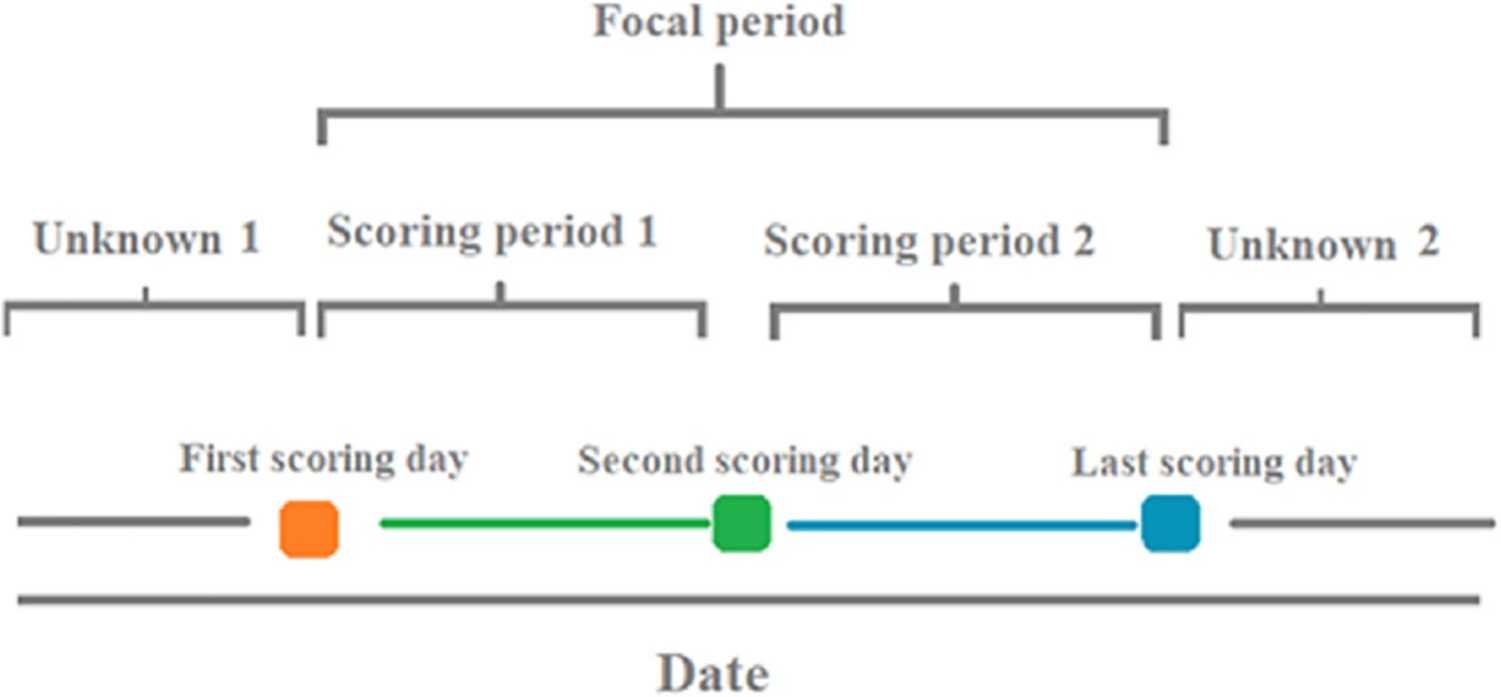
Timeline diagram of how focal periods were selected and how camera data was validated against injury / health scoring. Coloured squares indicate separate scoring events. The period of time between the first and last scoring event was identified as the focal period, and the space of time between consecutive scoring events were referred to as the scoring periods. The end of the batch (Unknown 2) was removed as no further injury scoring was gathered in order to validate the tail posture changes during this time. The start of the batch (Unknown 1) was removed due to variable data quality at the start of some batches.

Camera data on tail posture were summarised into daily proportions of each tail posture, and injury/health scores were only available once every ~14 days. For analysis, the injury/health scores were allocated to the period preceding a scoring event. The logic of this is that if harmful behaviours take place during a 14-day period and affect tail postures, the evidence of those behaviours would be apparent in the injury scoring at the end of that time. This is shown diagrammatically in Fig 6. Once the data were trimmed down to the focal period, the remaining data were checked to determine whether there were less than 20% of days within the focal period in the 41 batches were affected by low image count days (fewer than 30 images) or other missing data.

### Statistical methodology

All statistical tests and modelling were conducted in R version 4.0.2. Variables were correlated using Spearman’s rho (ρ) in base R. Linear mixed effect models were carried out using the *lme4* package (version 1.2.23). Stepwise regression was carried out using the *caret* package (version 6.0.86). Data accompany the paper in S2 File.

#### Null models

A series of linear mixed effect models using the proportion of low, mid or high tails as the response variable. The variation in the proportion of tail postures was modelled with fixed effects, which included environmental or husbandry effects (floor type, tail length, enrichment), temporal effects (days in the pen [covariate]), or technical effects (scorer identity, number of cameras, and tail image count [covariate]). Farm and batch were entered as random effects. Variables that did not significantly contribute to the variation in tail posture were dropped from the model.

#### Injury/health scoring models

Each of the injury and health scores were added to the null model in turn, and statistical improvement to the null model was assessed by the significance of change in model fit (p value) and an increase in the R^2^.

## Results and discussion

Injuries from social aggression affected around half the pigs, while more than half had some sign of at least minor ear (70%) or tail (65%) damage. Flank biting was rare affecting fewer than 5% of pigs. Injuries were generally minor, and the number of pigs with visible wounds on tails or ears was low. Lameness affected around 8% of pigs while over 80% had some ocular discharge.

### Correlations between variables

Tail postures showed low to moderate correlations. Tail angles were all significantly (p<0.001) negatively correlated with one another (Tail high–tail mid ρ = -0.28; Tail mid–tail low ρ = -0.23), with the proportion of tails high and proportion low showing the strongest negative relationship (ρ = -0.84, p<0.001). To fully understand the relationship between health and injury scores and tail postures, we ran a separate series of models using each of these three tail postures as the response variable.

Correlations between injury and health scoring variables are shown in Table 3. Within tail scores, the highest correlations are between the high tail injury and weighted total tail injury severity, showing the contribution of high scores to that metric. Low tail injury freshness (scabs) was highly correlated with high tail injury (scratches and wounds) as more minor low tail injuries (flattened or red tails) would not have been scored as scabs or wounds at all. Tail length loss is a cumulative indicator of tail injury, which indicates tail biting at some point in the past (a trailing indicator), and was only weakly correlated with other tail injury indicators. The exception to this is tail swelling, another trailing indicator, although swelling was also moderately correlated with low tail freshness (scabs) and high tail injury (scratches and wounds). The weak correlation between tail length loss and other tail injury metrics was somewhat unexpected. We carried out further correlation matrix analysis of docked and undocked pigs’ data separately. This revealed that for undocked pigs, tail length loss was more strongly correlated (all p<0.001) with high tail injury (r = 0.39), weighted total tail injury severity (r = 0.46) and low tail injury freshness (r = 0.52), while docked pigs showed weakly negative and non-significant associations for each of these. This suggests that tail biting injuries were associated with tail length loss in undocked pigs, but not in docked pigs, who had shorter tails and so less tail length to lose.

**Table 3:** Correlations between health and injury scores used in statistical modelling.

		Tail							Flank	Ear				Body	Lame
		Low tail injury severity	High tail injury severity	Weighted total tail injury severity	Low tail fresh-ness	High tail fresh-ness	Tail length loss	Tail Swelling	Flank injury severity	Low ear injury severity	High ear injury severity	Low ear fresh-ness	High ear fresh-ness	Body lesions	Lame-ness
**Tail**	Low tail injury severity														
	High tail injury severity	-0.02													
	Weighted total tail injury severity	0.39***	0.87***												
	Low tail injury freshness	-0.02	0.88***	0.79***											
	High tail injury freshness	-0.01	0.46***	0.34***	0.10										
	Tail length loss	-0.04	0.13	0.15*	0.18*	0.11									
** **	Tail swelling	-0.06	0.36***	0.38***	0.38***	0.17*	0.52***								
**Flank**	Flank injury severity	-0.16*	0.26***	0.22**	0.31***	-0.03	-0.03	0.17*							
**Ear**	Low ear injury severity	0.52***	0.25**	0.51***	0.26***	0.05	-0.04	0.05	0.11						
	High ear injury severity	-0.02	0.04	0.02	0.06	0.05	0.01	0.14	-0.01	0					
	Low ear freshness	-0.25**	-0.02	-0.18*	-0.11	0.19*	-0.06	-0.05	-0.09	-0.21**	0.03				
** **	High ear freshness	-0.11	-0.18*	-0.23**	-0.28***	0.08	-0.18*	-0.12	-0.03	-0.14	0.09	0.47***			
**Body**	Body lesions	0.24**	-0.11	0.04	-0.17*	0.03	-0.02	-0.15	0.19*	0.42***	-0.12	-0.09	-0.02		
**Lame**	Lameness	-0.20**	-0.15	-0.23**	-0.20**	0.08	0.17*	0.04	0.09	-0.15*	-0.03	0.05	-0.09	0.23**	
**Eyes**	Ocular discharge	-0.15	-0.08	-0.14	-0.15	-0.05	-0.09	0.02	-0.14	-0.03	-0.03	0.19*	0.10	0.15	0.22**

Spearman’s rank correlations (ρ) are shown, with asterisks to indicate statistical significance

* p<0.05

** p<0.01

*** p<0.001. Data from scoring days were used n = 166.

Within ear scores, high and low ear freshness were positively correlated, indicating that where ear injury is occurring, both scabs (low ear freshness) and fresh scratches and wounds (high ear freshness) occur in the group.

Between the scoring locations some correlations suggest that injuries occurred together. Most notably, low level ear injuries (scratches and ear damage) were positively associated with low, high and weighted total tail injury severity. These moderate correlations were higher than the weak to moderate positive associations previously reported between ear and tail biting lesions [30]. Flank injury was weakly positively associated with high tail injury severity (scratches and wounds) and low tail injury freshness (scabbed tails), again these associations were higher than previously reported [30]. Body lesions caused by social aggression showed a moderate positive correlation with low ear injury severity and were also weakly correlated with low tail injury severity. Lameness and ocular discharge showed only weak correlations with other injury scores.

### Linear models

Primary evaluation of the null models found that once the random effect of farm and batch had been accounted for, the model was not improved by inclusion of the fixed effect of floor type, tail length, or enrichment. Therefore, all null models had the same structure of farm and batch as the random effect, and days in the pen as a covariate. Since environmental or pig effects did not explain tail posture variation, it remains unclear why it varied so much between different farms and batches, including subsequent batches in the same pen. The varying experiences, behaviour and health of groups of pigs before they were first scored for the project might have caused these differences. Also, there was some variation in when the first scoring visit took place, relative to the day on which pigs had been moved into the pen. In our previous work [5], we had found that low tails increased for a few days when pigs were moved between pens. Finally, if damaging, stressful behaviour such as tail biting had begun prior to the pigs moving into the pen (tail biting can occur in weaners [35]), this might have contributed to variation between pens at the start of our study.

**Fig 7:**
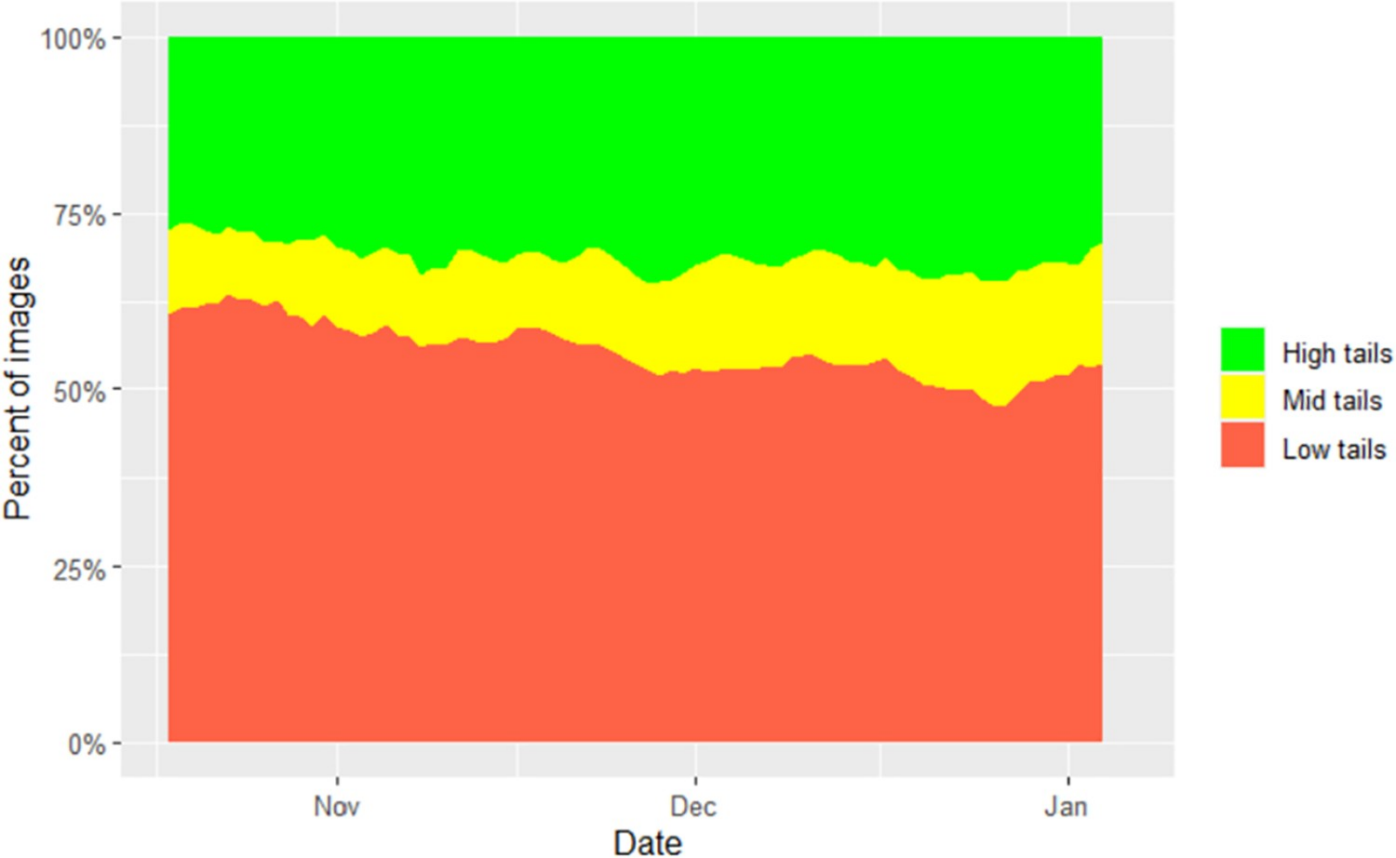
Example of tail posture data for a typical batch- stacked bar graph of proportion of low (red), mid (yellow) and high (green) tails by day.

Null models revealed that the percentage of low tails had an intercept of 54.86 (95% CI 49.12–60.59, p<0.001) and showed a decline with days in pen with a gradient estimated at -0.16 (-0.17 –-0.14), p<0.001). The percentage of mid tails had an intercept of 23.79 (21.17–26.41, p<0.001) and also declined slightly with days in pen at -0.05 (-0.06 –-0.03, p<0.001). The percentage of high tails had an estimated intercept of 21.49 (16.17–26.81, p<0.001) and increased with days in pen with an estimated gradient of 0.20 (0.18–0.22, p<0.001). Similar age effects on tail posture, with an increase in curly tails and a reduction in tucked tails with age, have recently been reported [36]. These trends are illustrated by data for a typical batch shown in Fig 7.

**Fig 8:**
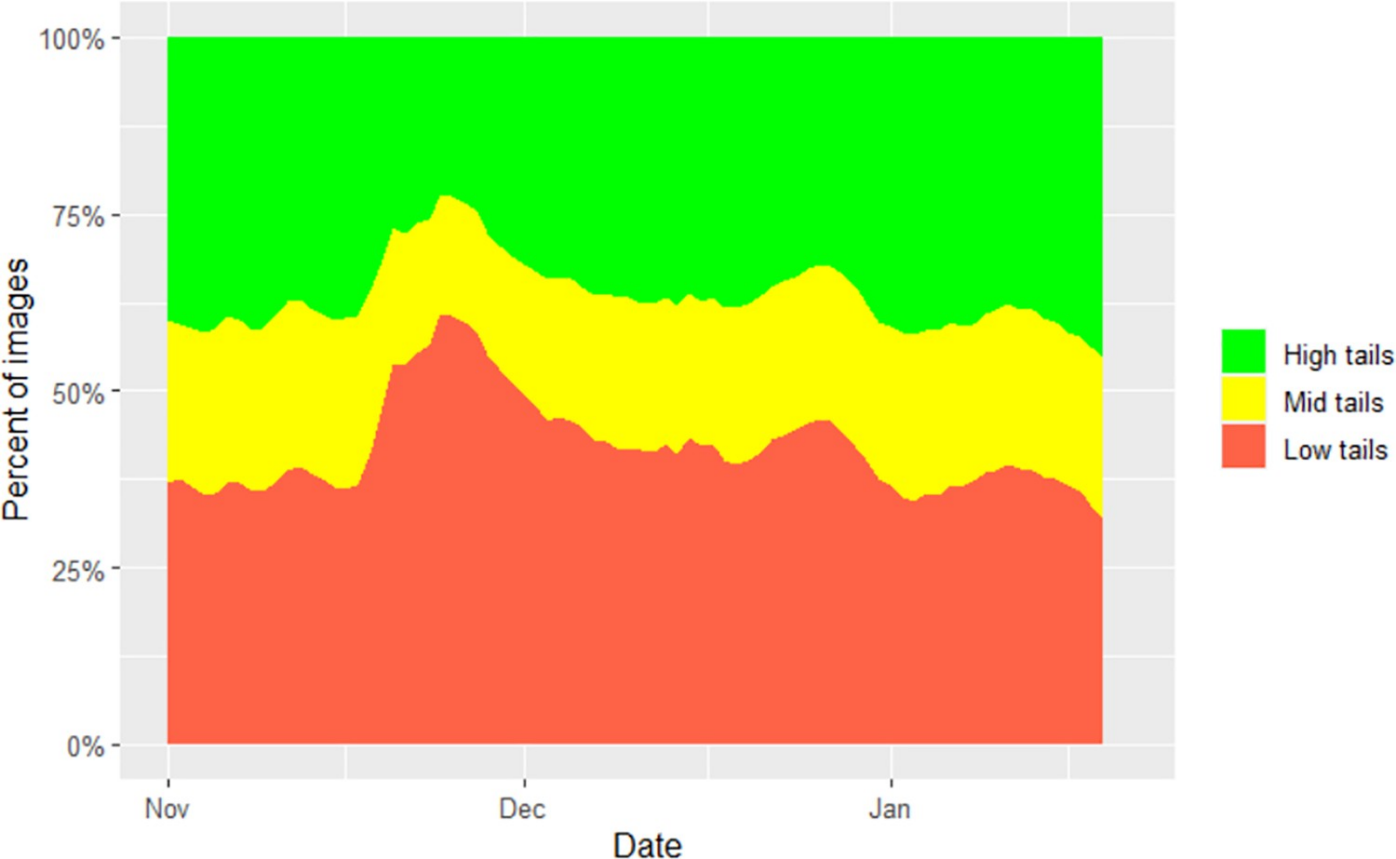
Example of tail posture data for a batch in which tail injury disrupted tail posture-stacked bar graph of proportion of low (red), mid (yellow) and high (green) tails by day.

Once the effects of farm, batch and days in pen in the null model are adjusted for, adding the health / injury scores each in turn to the null model found a number of significant effects (Table 4). Tail biting injuries were associated with tail posture in various ways that were consistent with our prediction [5] that increasing tail injury is associated with decreasing high tails and increasing low tails. An example of a batch in which tail posture was disrupted by tail injury is shown in Fig 8. Overall tail injury severity, a worsening of this metric between scoring periods, and tail length loss were associated with an increase in the proportion of low tails and a reduction in high tail posture. Of these, increasing weighted total tail injury was the most significant. Low tail injury severity was associated with reduced high tails and an increase in mid tails. Tail swelling was associated with more low tails. The proportion of pigs scored with severe tail injury was not associated with tail posture. There could be a number of reasons why the effects of tail biting on tail posture in this study might have been less apparent than in our previous work [5]. In the current study, the level of tail biting was a lot lower. Tail docking was prevalent, and the commercial pig producers in the study managed tail biting well, rather than deliberately orchestrating tail biting using high risk housing and management for experimental purposes [5]. Scoring every two weeks, rather than three times a week [5] also meant it was more difficult to track the time course of tail biting, making it harder to identify a clear association with tail posture. Anecdotally, a sharp reduction in high tails and an increase in low tails could sometimes be seen at the onset of an outbreak, before returning to baseline while tail injuries continued to accumulate. Another issue is that we suspect tail posture changes occur most rapidly at the onset of a tail biting outbreak, before recovering. If tail biting was already occurring before our first project visit, this could weaken the relationship between tail injury and tail posture observed.

**Table 4:** Results of linear mixed models of tail posture by injury and health scoring metrics.

		Proportion of Low Tails		Proportion of Mid Tails		Proportion of High Tails	
Injury/ health	Variable	Estimated effect (95% CI)	R^2^	Estimated effect (95% CI)	R^2^	Estimated effect (95% CI)	R^2^
**Tail**	Low tail injury severity	0.01 (-0.01–0.03)	0.807	0.02** (0.01–0.04)	0.699	-0.03** (-0.05 - -0.01)	0.820
	High tail injury severity	0.01 (-0.01–0.03)	0.808	-0.01 (-0.02–0.01)	0.694	0.00 (-0.02–0.01)	0.815
	Weighted total tail injury severity	0.02* (0.00–0.05)	0.811	0.01 (-0.01–0.02)	0.694	-0.03* (-0.05 - -0.01)	0.821
	Increasing weighted total tail injury	1.08*** (0.44–1.71)	0.807	-0.08 (-1.33–1.17)	0.693	-0.90** (-1.53 - -0.27)	0.815
	Low tail injury freshness	0.01 (-0.01–0.03)	0.807	-0.00 (-0.01–0.01)	0.693	-0.01 (-0.03–0.01)	0.816
	High tail injury freshness	-0.01 (-0.05–0.04)	0.806	-0.03* (-0.06–0.00)	0.699	0.04 (-0.00–0.09)	0.814
	Tail length loss	0.07** (0.02–0.12)	0.816	0.00 (-0.04–0.03)	0.694	-0.07* (-0.12 - -0.02)	0.821
	Tail swelling	0.05* (0.01–0.09)	0.809	-0.01 (-0.04–0.01)	0.696	-0.04 (-0.08–0.00)	0.815
**Flank**	Flank injury	-0.13*** (-0.19 - -0.08)	0.814	-0.02 (-0.06–0.02)	0.695	0.16*** (0.10–0.21)	0.824
**Ears**	Low ear injury severity	0.02 (0.00–0.04)	0.810	0.00 (-0.01–0.02)	0.692	-0.02* (-0.04 - -0.00)	0.819
	High ear injury severity	0.34 (-0.01–0.69)	0.808	0.06 (-0.18–0.30)	0.693	-0.40* (-0.75 - -0.05)	0.816
	Low ear injury freshness	-0.02 (-0.05–0.00)	0.805	0.01 (-0.01–0.02)	0.690	0.02 (-0.01–0.04)	0.814
	High ear injury freshness	0.07*** (0.03–0.11)	0.809	-0.13 (-0.15 - -0.10)	0.740	0.06** (0.02–0.09)	0.817
**Body**	Shoulder/body lesions	0.04*** (0.02–0.06)	0.814	-0.01 (-0.02–0.00)	0.693	-0.03** (-0.05 –-0.01)	0.820
**Lameness**	Lameness	0.14*** (0.10–0.17)	0.817	-0.05*** (-0.07 - -0.02)	0.694	-0.09*** (-0.12 - -0.06)	0.820
**Eyes**	Ocular discharge	0.06*** (0.03–0.09)	0.808	-0.12*** (-0.14 - -0.10)	0.724	0.06*** (0.02–0.09)	0.817

Null model included farm and batch as random effects and days in pen as a covariate. Null model R squared values were: Low tails R^2^ = 0.806, Mid tails R^2^ = 0.693, High tails R^2^ = 0.815.

Tail posture was also significantly affected by injury (other than tail injury) and ill health. An increasing proportion of lame pigs and also pigs with body lesions caused by social aggression were associated with an increase in low tails and a reduction in high tails, and more pigs with low or high ear injury severity were each associated with a reduction in high tails in the pen. These findings were consistent with the hypothesis that tail posture could be used as a more general indicator of pig welfare [27, 28]. The 3D machine vision system in the present study used a series of static ‘snapshot’ pig detections at the group level, so tail movements of any individual pig could not be captured. Tail movements have been suggested to provide information about pig welfare [28], which is an area for potential further refinement of our system and for a further validation study.

Ocular discharge was associated with increased low tail posture and high tail posture, and a decline in mid tail posture. Dark discharge below the eye, also known as ‘tear staining’ in pigs had been thought to be associated with disease such as atrophic rhinitis or a response to poor air quality [33]. More recently, studies in healthy pigs show that ‘tear staining’ may be a general indicator of negative welfare, as it can be induced by psychological stressors such as social isolation and lack of enrichment, and is associated with other stress indicators such as high plasma cortisol and eosinophils [37]. Farm studies have found a weak positive association between tear staining and tail and ear damage [33, 38]. Our tail posture results are therefore puzzling. If ocular discharge/tear staining indicates negative welfare, we might have expected not just an increase in low tails, but a decline in high tails too. Scores showing high ear injury freshness were also associated with an increase in both low and high tail postures in the pen.

Even more puzzling are our results on the relationship between flank biting injuries and tail posture: high levels of flank injuries were associated with more high tails and fewer low tails. This is the opposite of the predicted direction of effect if tail posture is a general welfare indicator assuming that being a recipient of all these forms of injury cause pigs some degree of distress and pain. Flank injury was not correlated with other forms of injury, except for a weak positive correlation with high tail injury severity.

Since tail posture can be influenced by injury and ill health other than tail biting, it does not appear to be as specific an indicator of tail biting as we had previously thought [5]. Some other previously suggested indicators (activity, feeder and drinker visits) are likely to be non-specific too, as they can be affected by many things. It may be that to more accurately predict tail biting, specific behaviours (such as tail-in-mouth), or behaviours used in combination with tail posture change [39, 40] should be used. Other possible ‘machine vision’ approaches to the automation of tail biting detection include identification of the specific movements of tail biting itself [41] or changes in group-level pig movements characterised by optical flow [42].

Our study has a number of limitations which could be improved on in further work. The use of twice-weekly scoring means we could not capture the rapid escalation of outbreaks of tail, ear or flank biting. Instead, available staff time was utilised across multiple sites, in order to improve the robustness of results across variable farm systems. The large variation between farms in pen size meant that number of pigs per camera was highly variable. As the cameras cover a similar floor area, the proportion of the pen covered by cameras was much less in larger pens. We had no control over which pigs were under the cameras and when, and we are likely to over-sample pigs that spend more time at or near the feeders. All of these factors are likely to have contributed to the between-farm variation.

Another really interesting issue for this work and for others is the question of group vs individual animal analysis. Here, our camera system worked at a group level as a series of snapshots, did not track individual pigs moving within the frame, and had no way to identify an individual pig leaving the frame and returning. We have further data on pigs under our 3D weight and tail cameras, collected on farms where individual pig identity is known due to radio-frequency identity (RFID) ear tags and readers in the pen. We expect this to provide further insights, in particular the relationship between tail and other injury and health scores and tail posture at an individual pig level.

## Conclusion

This study adds to a growing body of work where machine vision methods are used to automate the detection of aspects of pig behaviour [3, 43–46] and extends this concept, demonstrating an important step change by developing and validating its use on commercial farms over an extended period for the first time.

Our findings bring to mind the opening sentences of Anna Karenina (Leo Tolstoy 1877) “All happy families are alike; each unhappy family is unhappy in its own way” [47]. Relationships between tail injury and tail postures were as expected, but other forms of injury and ill-health also impacted on tail posture. Healthy batches of pigs showed trends in tail posture with age (“All happy families are alike”), and pigs with signs of lameness, ear-, flank- or tail-biting showed deviations of various kinds from this general time trend. As such, any deviation from this expected tail posture trend suggests that something could be wrong and pigs should be inspected more closely to identify specifically what that is (“each unhappy family is unhappy in its own way.”). These deviations in tail posture therefore have the potential to be used as the basis of a precision livestock farming warning system for pig producers.

## Supporting information

S1 File Injury and health scoring system. (DOCX)

S2 File Modelling dataset TailTech. (XLSX)
