# Supplementary material for: Changes in tail posture detected by a 3D machine vision system are associated with injury from damaging behaviours and ill health on commercial pig farms
Source: PLoS One. 2021 Oct 28;16(10):e0258895. doi: 10.1371/journal.pone.0258895 (PMC8553069; doi:10.1371/journal.pone.0258895)
Supplement: S1 File — (DOCX) [file pone.0258895.s001.docx]

***S1 Injury and health scoring system***

The following descriptors for scoring of injury to tails, flanks, ears and body were used. Lameness was also scored and nasal and occular discarge were each scored as present or absent.

**Tail injury** – damage to tails caused by tail biting

*Severity of injury*

**0** No tail damage

**1** Flattened - tail is not round, feels flattened like it has been sucked or chewed

**2** Red – general redness or red marks, no broken skin

**3** Bite marks or scratches – not just red, can see the puncture wounds

**4** Wound – Raw flesh, chewed, damaged tail

*Freshness of injury*

**0** No Wound

**1** Intact scab or healed old wound.

**2** Not intact scab – older blood, red tissue

**3** Fresh bite/scratch or wound, not bleeding or weeping

**4** Fresh bite/scratch or wound – weeping but not bleeding (can include bloodied, blood stuck to tail hair)

**5** Fresh bite/scratch or wound – bleeding – dripping with blood & splattering own bottom or other pigs.

*Tail Length (for undocked pigs)-* note that tail length should be scored using the living flesh part of the tail. If there is necrotic tissue at the end of the tail which will fall off, then it shouldn’t be counted as part of the tail length- i.e. these pigs should score at least 1 or more.

**0** Full length tail – has the fluffy bit on the end

**1** Shortened tail – fleshy end, shorter than average

**2** More than half the tail missing

**3** <1 cm left of the tail – tail end almost flush with the rump

*Tail Length (for docked pigs)- as above for necrotic tails.*

**0** Full length tail – tail remains as long as when it was docked (compared to others in batch or pen)

**1** Shortened tail – fleshy end, shorter than average

**2** Tail is less than half the length it was docked to

**3** <1 cm left of the tail - tail end almost flush with the rump

*Tail Swelling*

**0** No swelling

**1** Swelling, tail looks swollen, creases in curves, pronounced or hacked

**Flank Injury** - caused by flank biting

*Severity of injury*

**0** No Damage

**1** Red/Minor Scratches

**2** Damaged skin

**3** Wound – raw flesh

*Freshness of injury*

**0** No Wound

**1** Intact scab or healed old wound

**2** Not intact scab – older blood, red tissue

**3** Fresh bite/scratch or wound, not bleeding or weeping

**4** Fresh bite/scratch or wound – weeping or bleeding (bloodied)

*Size of injured area*

**0** No wound

**1** Wound or scab area is <2 cm x 2cm

**2** Wound or scab area is >2cm x 2cm

**Ear injury** caused by ear biting – Score left ear & right ear

*Severity of injury*

**0** No Damage

**1** Red/Minor Scratches

**2** Damaged skin

**3** Wound – raw flesh

*Freshness of injury*

**0** No Wound

**1** Intact scab or healed old wound

**2** Not intact scab – older blood, red tissue

**3** Fresh bite/scratch or wound, not bleeding or weeping

**4** Fresh bite/scratch or wound – weeping or bleeding (bloodied)

*Size of injured area*

**0** No damage

**1** Top or bottom of ear damaged

**2** Top and bottom of ear damaged

**3** More than half of ear circumference shows signs of damage

Swelling/Fluid Filled

**0** No swelling

**1** Swelling

**2** Unrelated fluid filled ear

**Skin lesions** – pigs with body marks caused by fighting- these are principally scratch marks to neck/shoulders.

**0** No Lesion - Pigs without any of the below body marks

**1** Mild - Pigs with mild body marks. Linear lesion longer than 10cm or if there are 3 or more 3cm lesions or if there is a circular area larger than 1cm diameter

**2** Severe - Pigs with severe body marks. Lesion is larger than 5x5cm diameter, or lesion extends into deeper layers of skin, or lesions cover a large percentage of skin (>25%)

**Lameness** – pigs showing altered gait

**0** Not Lame – gait appears balanced and normal

**1**  Lame – altered gait (limp) while moving but still placing weight on all 4 feet

**2** Severely Lame – pig places no or very little weight on affected limb, and/or is reluctant to rise from lying.

**Nasal/Ocular Discharge** – mucus or dark discharge at the nostrils or below the eyes (scored separately)

**0** Not Present

**1** Present
